# Guiding Sole Intraoperative Radiotherapy in Breast Cancer According to ASTRO Guidelines: Mitigating Adverse Outcomes in a Taiwan Single‐Center

**DOI:** 10.1002/cam4.70537

**Published:** 2024-12-30

**Authors:** Hsin‐Yi Yang, Yuk‐Wah Tsang, Chi‐Wen Tu, Yu‐Chen Hsu

**Affiliations:** ^1^ Clinical Data Center Ditmanson Medical Foundation Chia‐Yi Christian Hospital Chiayi City Taiwan; ^2^ Department of Radiation Oncology Ditmanson Medical Foundation Chia‐Yi Christian Hospital Chiayi City Taiwan; ^3^ Department of Biomedical Engineering Chung Yuan Christian University Taoyuan City Taiwan; ^4^ Department of Surgery Ditmanson Medical Foundation Chia‐Yi Christian Hospital Chiayi City Taiwan

**Keywords:** breast cancer, intraoperative radiotherapy, whole‐breast external beam radiotherapy

## Abstract

**Background:**

Intraoperative radiotherapy (IORT) is considered a de‐escalating adjuvant treatment for breast cancer low‐risk patients. However, the broader criteria applied by the Taiwan IORT Study Cooperative Group led to an increased rate of locoregional recurrence (LRR) among patients receiving only IORT. Consequently, we revised the criteria for sole IORT treatment to include patients who meet the American Society for Radiation Oncology (ASTRO) eligibility standards. This study aims to investigate how aligning treatment strategies with ASTRO guidelines impacts oncological outcomes in patients receiving IORT.

**Methods:**

From September 2014 to March 2022, a retrospective review of 632 patients with invasive breast cancer undergoing breast‐conserving surgery assessed outcomes following External Beam Radiation Therapy (EBRT), sole IORT, or IORT combined with supplemental EBRT (IORT+boost), with a mean follow‐up period of 4.71 ± 2.10 years. Strategic modifications following the ASTRO guidelines were implemented in April 2021 for sole IORT recipients.

**Results:**

ASTRO‐suitable patients had significantly lower rates of LRR (0.00% vs. 10.64%), distant metastasis (0.00% vs. 4.00%), and any recurrence (0.00% vs. 7.79%) than non‐suitable patients (*p* < 0.05). After adjusting for confounders, the overall and non‐suitable patients in the IORT group both exhibited significantly higher hazard ratios for LRR than those in the EBRT group, with values of 5.79 (95% confidence intervals (CI) = 1.75–19.18) and 5.48 (95% CI = 1.66–18.02), respectively.

**Conclusions:**

Aligning treatment criteria with ASTRO guidelines for sole IORT significantly reduces locoregional recurrence rates in patients with invasive breast cancer, highlighting the efficacy of these strategic modifications in improving oncological outcomes.

## 1 Introduction

Adjuvant radiotherapy following breast‐conserving surgery is pivotal for mitigating the rates of recurrence rates and breast cancer‐related mortality in the long term [1]. Administering radiotherapy to the preserved breast reduces the disease recurrence rate by 50% and decreases the breast cancer mortality rate by approximately 16.67% [2]. While External Beam Radiation Therapy (EBRT) remains the standard option, a shorter course of radiotherapy has been developed to enhance efficiency and reduce the treatment duration [3, 4]. Intraoperative radiotherapy (IORT) is increasingly acknowledged as a de‐escalating adjuvant therapy for low‐risk breast cancer patients [5, 6]. Sole IORT represents a focused treatment option during surgery, significantly reducing radiation exposure to vital organs such as the heart and lungs [5, 6]. The emphasis on IORT's appeal within the medical community lies in its recognition as a meticulously calibrated risk‐tailored treatment, underscored by influential phase III trials such as TARGIT and ELIOT [5, 6]. The American Society for Radiation Oncology (ASTRO) guidelines emphasize several key recommendations for the use of IORT in breast cancer treatment, including counseling patients regarding the higher risk of ipsilateral breast tumor recurrence with IORT compared with whole‐breast irradiation. This highlights the need for prospective monitoring of long‐term local control and toxicity with low‐energy radiograph IORT given the limited follow‐up period, and the restriction of IORT to women with invasive cancer is considered “suitable” [7].

In Taiwan, the Taiwan IORT Study Cooperative Group (T‐IORTSCG) criteria are significantly less stringent than the ASTRO guidelines in regulating the use of sole IORT [8]. Per the 2017 study conducted by the T‐IORTSCG, a low locoregional recurrence (LRR) rate of 0.8% was observed over an average follow‐up period of 1.3 years [8]. A meta‐analysis revealed a higher risk of local recurrence associated with IORT than with EBRT, particularly in studies with long‐term follow‐up or those published after 2020 [9]. In 2021, unfavorable long‐term outcomes associated with the T‐IORTSCG IORT criteria were published [10]. The observed risk of LRR was 10.64% for IORT, which was significantly higher than the 2.44% reported for EBRT with a mean follow‐up period of 3.53 years. Notably, hazard ratios (HRs) for LRR were particularly elevated in patients deemed unsuitable per the ASTRO criteria and those under 50 years of age [10].

Following the compelling findings of 2021, the criteria for sole IORT in our institute were revised from T‐IORTSCG to ASTRO criteria. Nevertheless, understanding the profound impact of strategic modifications in the application of IORT warrants more in‐depth exploration. This study aimed to assess oncological outcomes following the implementation of strategic modifications, per the ASTRO guidelines, in patients undergoing sole IORT.

## 2 Methods

### 2.1 Study Population and Design

A retrospective cohort study was performed using data from the electronic medical record database [Ditmanson Research Database (DRD)] at the Ditmanson Medical Foundation Chia‐Yi Christian Hospital, a regional hospital in southern Taiwan. This study was approved by the Research Ethics Committee of Ditmanson Medical Foundation Chia‐Yi Christian Hospital, Taiwan (CYCH‐IRB No.: 2023029). The Institutional Review Board waived the requirement for informed consent due to the retrospective design and use of anonymized clinical data. All methods were conducted in accordance with applicable guidelines and regulations. In this study, we included 688 patients with invasive breast cancer who underwent breast‐conserving surgery between September 2014 and March 2022. At our institute, multifocality and multicentricity of the tumors were assessed using breast ultrasound, mammography, and contrast‐enhanced chest CT scans. Additionally, 184 of the 632 patients underwent breast MRI exams. After excluding 56 patients who declined radiotherapy, 632 who received EBRT, sole IORT, or IORT with supplemental EBRT (IORT+boost) were included in the analysis. Clinicopathological characteristics and oncological outcomes, including patient characteristics, type of axillary surgery, tumor pathological results, type of adjuvant radiotherapy, type of concurrent treatment, type of recurrence, and survival status at the most recent follow‐up, were recorded.

The inclusion criteria for sole IORT, from September 2014 to March 2021, which were adapted from the T‐IORTSCG criteria, were negative resection margin, unifocal invasive tumor measuring less than 3 cm, no evidence of lymph node involvement, and a minimum age of 40 years. Since April 2021, the eligibility criteria for sole IORT have been revised to include a negative resection margin, unifocal invasive tumor measuring less than 2 cm, no evidence of lymph node involvement and lymphovascular invasion (LVI), positive hormone status, and a minimum age of 50 years. Patients not fulfilling all sole IORT criteria have been referred for supplemental EBRT since April 2021. All patients underwent extensive preoperative counseling from the surgeon. Radiation treatment options were explained to the patients, including standard EBRT and IORT. The detailed protocol for conducting EBRT and IORT using the Xoft Axxent eBx delivery system has been described in our previous study [10]. In brief, our institute required intraoperative frozen section confirmation of the absence of malignant cells in the sentinel lymph node and margin status before conducting IORT. A positive section margin is a contraindication for IORT. After breast‐conserving surgery, the tumor bed was mobilized. A chest wall shield, made of a pliable piece of lead, was temporarily placed into the cavity for the duration of radiation treatment to protect the underlying heart, ribs, and lungs from scattered radiation. Ultrasound was used to ensure that there was a distance of at least 10 mm between the surface of the applicator and the skin. A planned dose of 20 Gy to the balloon surface was delivered over 8 ± 1.5 min. After radiation treatment, the lumpectomy cavity was irrigated and closed in a standard manner.

Patients were categorized into distinct risk groups based on the ASTRO ABPI 2017 consensus [10]. Eligibility for IORT was determined if patients met all the following criteria: age above 50, negative resection margin, negative axillary lymph nodes, tumor size ≤ 2.0 cm, absence of LVI, and positive hormone status.

The EBRT regimen with or without regional nodal irradiation was offered at either conventional (50.0–50.4 Gy at 1.8–2.0 Gy) or hypofractionated (40.05–42.56 Gy in 15–16 fractions) regimen. An additional boost dose to tumor bed was delivered at either conventional (10.0–14.0 Gy in 5–7 fractions) or moderate hypofractionation (10.0 Gy in 4 fractions) regimens. IORT using the Xoft Axxent eBx delivery system delivered 20 Gy to the tumor cavity surface, with the X‐ray beam energy set at 50 kV. Supplementary EBRT was planned with a linear accelerator (Varian Clinac ix), mainly utilizing 6 MV X‐rays or a combination of 6 and 10 MV X‐rays depending on the patient's breast size. The supplementary RT dose ranged from 40 to 42.5 Gy in 15–16 daily fractions, according to the discretion of the radiation oncologist. Notably, no tumor bed boost was administered because cavity IORT had already served as a boost dose to the tumor cavity.

### 2.2 Outcomes

The primary endpoints of this study were LRR, overall survival, and breast cancer‐specific survival. Patients in the cohort were followed until (1) LRR, (2) death, (3) last contact before the end of June 30, 2023, or (4) the end of June 30, 2023.

### 2.3 Statistics

Quantitative variables were presented using mean values with standard deviations (for normally distributed data) or median values with interquartile ranges (for skewed data). Qualitative variables were presented as frequencies and percentages. An analysis of variance and chi‐square test or Fisher's exact test, as appropriate, were conducted to assess the distribution of patient characteristics, as well as clinical and pathologic characteristics among individuals who received treatment in the EBRT, IORT, and IORT+boost groups. To evaluate the cumulative risks of clinical outcomes for the three adjuvant radiotherapy groups, the Kaplan–Meier analysis was employed. The difference between the survival curves was examined using the log‐rank test. To investigate the associations between clinical outcomes and each clinical factor, HRs and 95% confidence intervals (CIs) were estimated using crude and adjusted Cox proportional hazard models, comparing the IORT and IORT+boost groups with the EBRT group. Subgroup analyses were conducted based on the ASTRO consensus statement risk groups to explore potential differences in response to different kinds of adjuvant radiotherapy. All statistical analyses were performed using the R programming language and environment (http://www.r‐project.org/). Two‐tailed *p*‐values of < 0.05 were considered statistically significant.

## 3 Results

### 3.1 Baseline Characteristics and Clinical Outcomes of the Study Population

**FIGURE 1:**
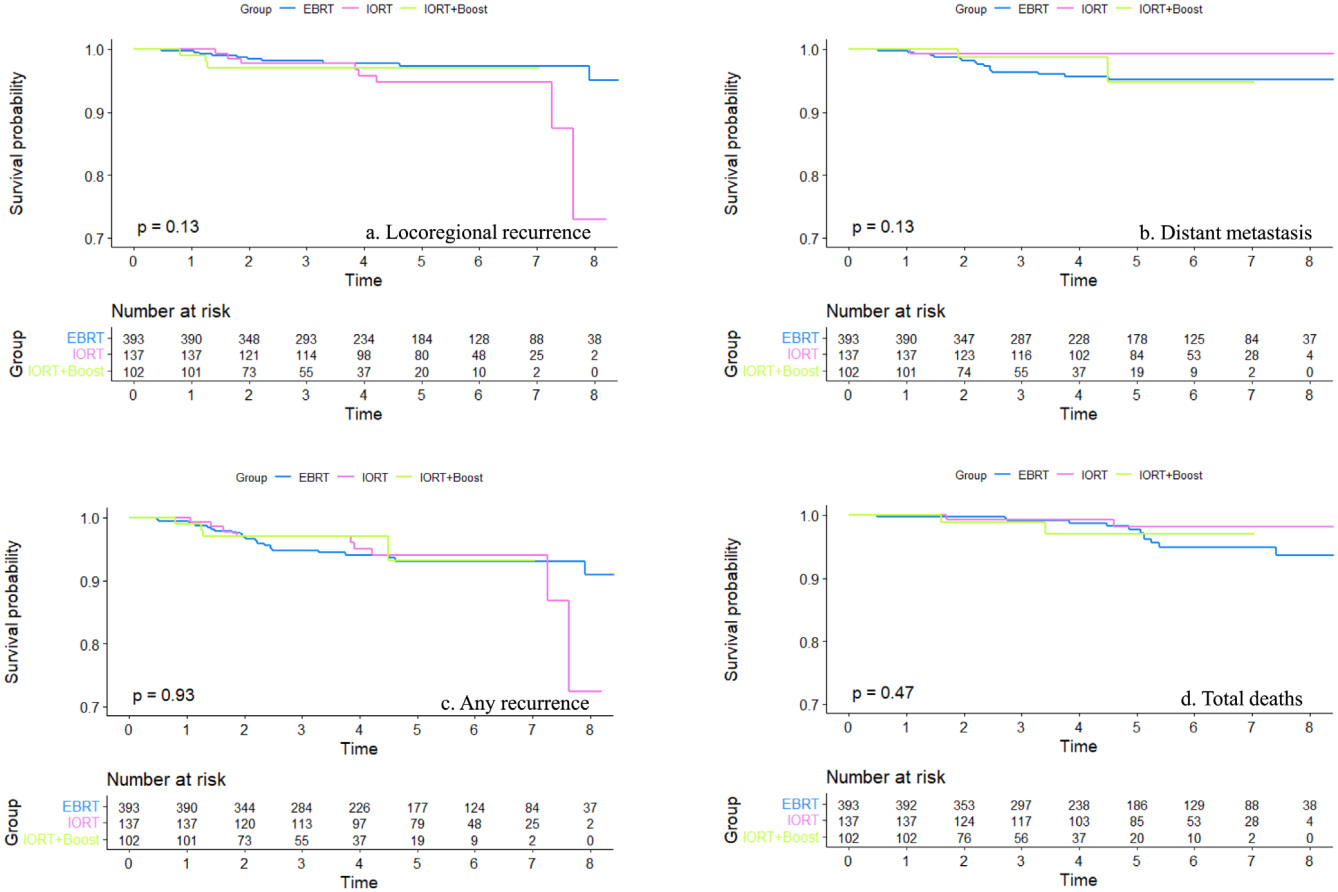
Univariable Kaplan–Meier curves in patients with breast cancer for (a) locoregional recurrence, (b) distant metastasis, (c) any recurrence, or (d) total deaths.

Table 1 presents the demographic characteristics of individuals who underwent treatment in the EBRT, IORT, and IORT+boost groups. The mean age in the IORT group (56.50 ± 10.57 years) was significantly higher than those in the EBRT (52.54 ± 10.50 years) and IORT+boost (54.89 ± 10.21 years) groups. Furthermore, statistically significant differences in pT stage, tumor size, number of examined lymph nodes, pN stage, proportion of LVI, proportion of chemotherapy, and proportion of target therapy were observed among the three groups (*p* < 0.05). However, no significant differences in BMI, section margin, ER/PR status, Her‐2 status, and hormone therapy were found among the three groups. In addition, the Kaplan–Meier curves, which depict the cumulative probability of LRR, distant metastasis, any recurrence, and total deaths, did not differ significantly among the three groups (log‐rank test, *p* > 0.05) (Figure 1).

**TABLE 1:** Demographic and clinical characteristics of patients.

	EBRT	IORT	IORT + boost	*p*
Number	393	137	102	
BMI	24.88 ± 4.06	24.75 ± 4.06	26.02 ± 4.35	0.188
≦ 18	8 (2.07)	4 (2.94)	1 (0.99)	
18–24	168 (43.41)	57 (41.91)	32 (31.68)	
> 24	211 (54.52)	75 (55.15)	68 (67.33)	
Age	52.54 ± 10.50	56.50 ± 10.57	54.89 ± 10.21	< 0.001
< 50	153 (38.93)	35 (25.55)	34 (33.33)	
50–59	131 (33.33)	45 (32.85)	30 (29.41)	
≧ 60	109 (27.74)	57 (41.61)	38 (37.25)	
pT‐stage				
T1	276 (72.63)	112 (81.75)	60 (60.61)	0.002
T2	104 (27.37)	24 (17.52)	39 (39.39)	
T3, 4	0 (0.00)	1 (0.73)	0 (0.00)	
Tumor size (mm)	17.74 ± 11.60	13.64 ± 7.04	18.87 ± 7.98	< 0.001
Section margin				
Negative	383 (97.95)	136 (100.00)	99 (98.02)	0.245
Positive	8 (2.05)	0 (0.00)	2 (1.98)	
Number of examed lymph node				
3–10	328 (83.46)	132 (96.35)	82 (80.39)	< 0.001
> 10	65 (16.54)	5 (3.65)	20 (19.61)	
pN‐stage				
N0	313 (80.88)	136 (99.27)	63 (63.00)	< 0.001
N1 + N2	68 (17.57)	1 (0.73)	36 (36.00)	
N3	6 (1.55)	0 (0.00)	1 (1.00)	
NA	5 (1.28)	0 (0.00)	0 (0.00)	
ER/PR status				
Negative	80 (20.36)	17 (12.41)	17 (16.67)	0.106
Positive	313 (79.64)	120 (87.59)	85 (83.33)	
Her‐2 status				
Negative	190 (48.35)	80 (58.39)	51 (50.00)	0.127
Positive	203 (51.65)	57 (41.61)	51 (50.00)	
Lymphovascular invasion				
Negative	217 (62.00)	112 (83.58)	42 (42.42)	< 0.001
Positive	133 (38.00)	22 (16.42)	57 (57.58)	
Risk group^a^				
Non‐suitable	306 (77.86)	76 (55.47)	93 (91.18)	< 0.001
Suitable	87 (22.14)	61 (44.53)	9 (8.82)	
Chemotherapy				
No	133 (33.84)	85 (62.04)	21 (20.59)	< 0.001
Yes (adjuvent)	215 (54.71)	52 (37.96)	73 (71.57)	
Yes (neoadjuvent)	45 (11.45)	0 (0.00)	8 (7.84)	
Hormone therapy				
No	86 (21.88)	22 (16.06)	18 (17.65)	0.278
Yes	307 (78.12)	115 (83.94)	84 (82.35)	
Target therapy				
No	341 (86.77)	134 (97.81)	87 (85.29)	0.001
Yes	52 (13.23)	3 (2.19)	15 (14.71)	
Follow‐up time	4.87 ± 2.16	5.21 ± 1.87	3.42 ± 1.61	< 0.001
Locoregional recurrence				
No	383 (97.46)	129 (94.16)	99 (97.06)	0.175
Yes	10 (2.54)	8 (5.84)	3 (2.94)	
Distant metastasis				
No	377 (95.93)	136 (99.27)	100 (98.04)	0.114
Yes	16 (4.07)	1 (0.73)	2 (1.96)	
Any recurrence				
No	369 (93.89)	128 (93.43)	98 (96.08)	0.649
Yes	24 (6.11)	9 (6.57)	4 (3.92)	
Total deaths				
No	381 (96.95)	135 (98.54)	100 (98.04)	0.547
Yes	12 (3.05)	2 (1.46)	2 (1.96)	
Cancer‐related death				
No	388 (98.73)	137 (100.00)	101 (99.02)	0.417
Yes	5 (1.27)	0 (0.00)	1 (0.98)	

Abbreviations: BMI, body mass index; EBRT, external beam radiation therapy; IORT, intraoperative radiation therapy.

^a^ Risk group = the ASTRO consensus statement risk groups.

### 3.2 Risk Factors for Clinical Outcomes

**FIGURE 2:**
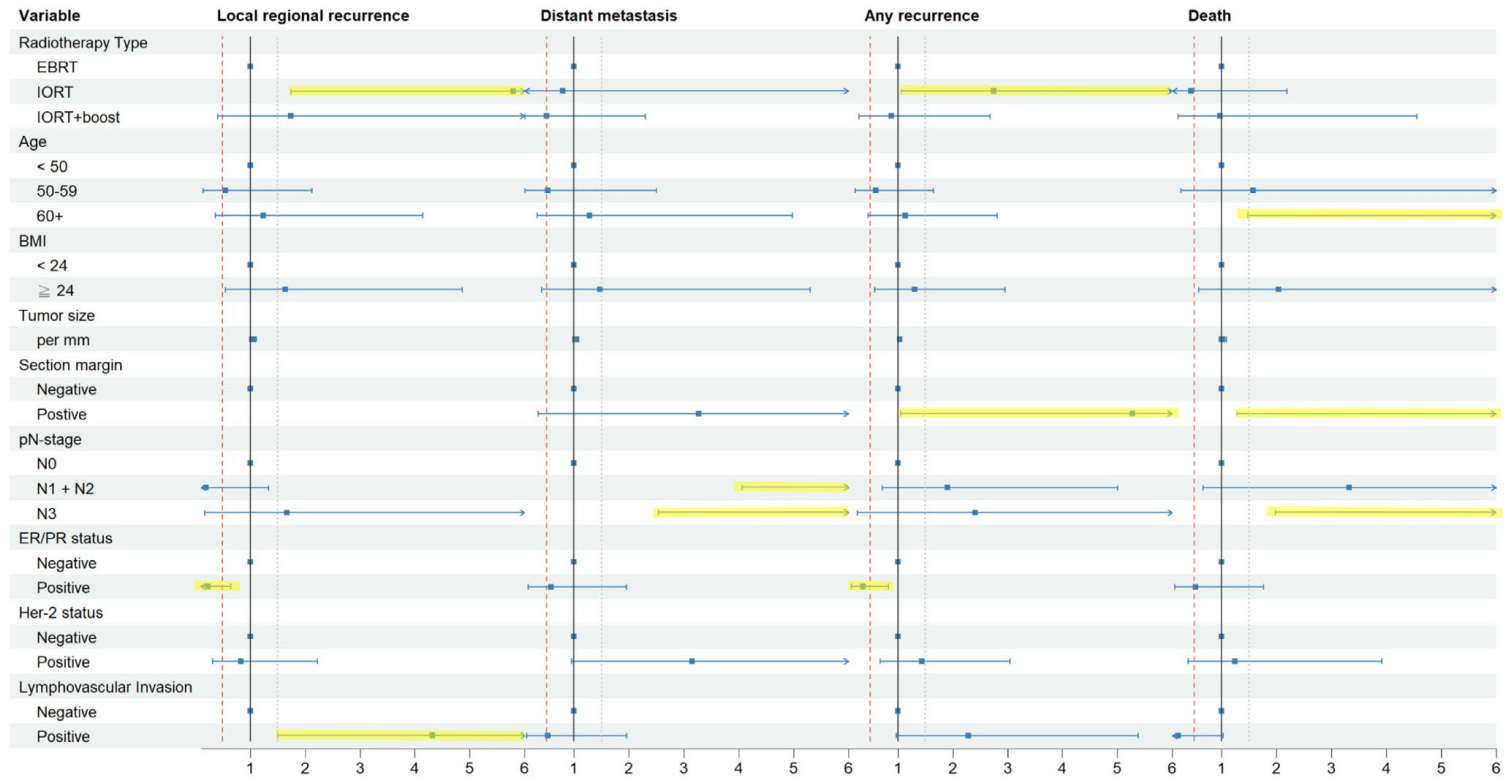
Cox proportional hazards regression multivariate analysis for associations between different types of adjuvant radiotherapy (external beam radiation therapy (EBRT), intraoperative radiation therapy (IORT), and IORT+boost) and locoregional recurrence, distant metastasis, any recurrence, and total deaths.

After adjusting for clinical and pathologic characteristics, patients undergoing IORT showed higher adjusted HRs (aHR) for both LRR and any recurrence compared with those receiving EBRT. The aHR for LRR was 5.79 (95% CI = 1.75–19.18, *p* = 0.004), and that for any recurrence was 2.76 (95% CI = 1.08–7.07, *p* = 0.035). However, no significant difference between IORT or IORT with boost and EBRT regarding overall mortality and distant metastasis after adjusting for clinical and pathologic characteristics was observed (*p* > 0.05). A resection margin had a significant positive association with any recurrence and LRR (aHR = 5.28; 95% CI = 1.07–26.15; *p* = 0.041 and aHR = 50.40; 95% CI = 6.66–381.66, respectively), whereas the ER/PR status demonstrated a significant inverse association (aHR = 0.38; 95% CI = 0.17–0.83; *p* = 0.015 and aHR = 0.23; 95% CI = 0.08–0.65; *p* = 0.005, respectively). Additionally, LVI (aHR = 4.23; 95% CI = 1.51–12.39; *p* = 0.006) was significantly associated with an increased risk of LRR after adjusting for potential confounders (Figure 2).

### 3.3 Association Between ASTRO Risk Groups and Clinical Outcomes

**FIGURE 3:**
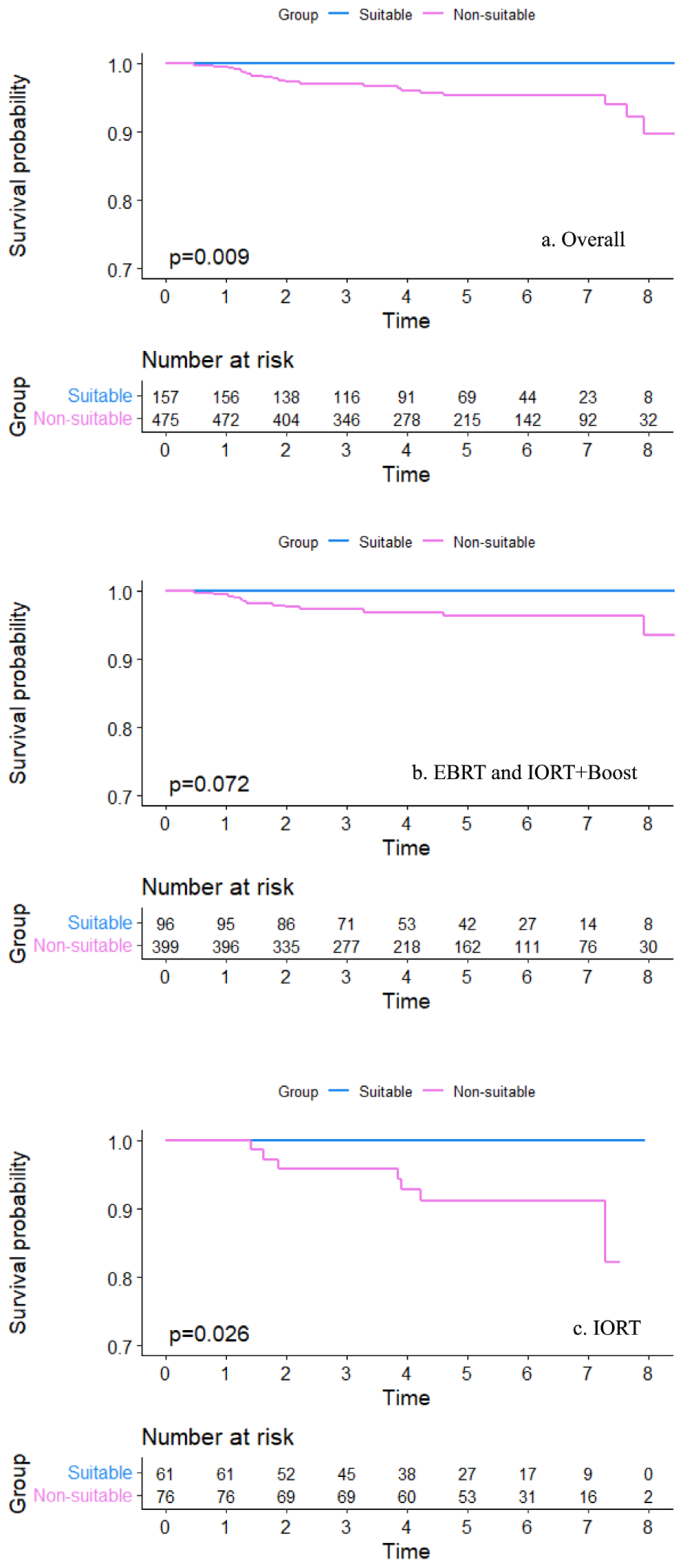
Cumulative incidence rates of local‐regional recurrence in suitable and non‐suitable groups for (a) overall patients; (b) patients treated with EBRT and IORT+Boost; (c) patients treated with IORT.

Our study examined the association between ASTRO consensus statement risk groups and clinical outcomes. The non‐suitable group showed significantly higher rates of LRR (10.64% vs. 0.00%), distant metastasis (4.00% vs. 0.00%), and any recurrence (7.79% vs. 0.00%) compared with the suitable group (*p* < 0.05). However, there were no significant differences in the rate of total or cancer‐specific deaths among the three groups (Table 2). The cumulative probability of LRR, as depicted by Kaplan–Meier curves (Figure 3), differed significantly between the suitable and non‐suitable groups (log‐rank test, *p* = 0.009). Notably, the IORT group showed significant differences (log‐rank test, *p* = 0.026), whereas no significant differences were found in the EBRT and IORT+boost groups (log‐rank test, *p* = 0.072).

**TABLE 2:** Association between the ASTRO consensus statement risk groups and clinical outcomes.

	Suitable	Non‐suitable	*p*
Locoregional recurrence			
No	157 (100.00)	454 (95.58)	0.015
Yes	0 (0.00)	21 (4.42)	
Distant metastasis			
No	157 (100.00)	456 (96.00)	0.023
Yes	0 (0.00)	19 (4.00)	
Any recurrence			
No	157 (100.00)	438 (92.21)	0.001
Yes	0 (0.00)	37 (7.79)	
Total deaths			
No	152 (96.82)	464 (97.68)	0.758
Yes	5 (3.18)	11 (2.32)	
Cancer‐related death			
No	157 (100.00)	469 (98.74)	0.347
Yes	0 (0.00)	6 (1.26)	

### 3.4 Association Between LRR and Different Adjuvant Radiotherapy in the Non‐Suitable Group

In the non‐suitable group, we found a significantly higher LRR rate in the IORT group compared with the EBRT and IORT+boost groups (*p* = 0.018). However, no significant differences were observed in terms of distant metastasis, total deaths, and cancer‐specific death among the three groups of non‐suitable patients (Table 3). After adjusting for clinical and pathologic characteristics, the non‐suitable group, whose members received IORT, had a higher HR for LRR than groups whose members received EBRT, with an HR of 5.48 (95% CI = 1.66–18.02, *p* = 0.005; Table 4).

**TABLE 3:** Association between different adjuvant radiotherapies (EBRT, IORT, and IORT + boost) and clinical outcomes in the non‐suitable group.

	EBRT	IORT	IORT + boost	*p*
Locoregional recurrence				
No	296 (96.73)	68 (89.47)	90 (96.77)	0.018
Yes	10 (3.27)	8 (10.53)	3 (3.23)	
Distant metastasis				
No	290 (94.77)	75 (98.68)	91 (97.85)	0.178
Yes	16 (5.23)	1 (1.32)	2 (2.15)	
Any recurrence				
No	282 (92.16)	67 (88.16)	89 (95.70)	0.191
Yes	24 (7.84)	9 (11.84)	4 (4.30)	
Total deaths				
No	298 (97.39)	74 (97.37)	92 (98.92)	0.675
Yes	8 (2.61)	2 (2.63)	1 (1.08)	
Cancer‐related death				
No	301 (98.37)	76 (100.00)	92 (98.92)	0.513
Yes	5 (1.63)	0 (0.00)	1 (1.08)	

Abbreviations: EBRT, external beam radiation therapy; IORT, intraoperative radiation therapy.

**TABLE 4:** Univariate and multivariate analyses for locoregional recurrence in the non‐suitable group.

	Crude HR (95% CI)	*p*	Adjusted HR (95% CI)^a^	*p*
EBRT	Reference		Reference	
IORT	3.13 (1.28–7.64)	0.012	5.48 (1.66–18.02)	0.005
IORT+boost	1.02 (0.29–3.53)	0.979	1.55 (0.37–6.49)	0.547

^a^ Adjusted: BMI, age, tumor size, section margin, pN‐stage, ER/PR status, Her‐2 status, lymphovascular invasion, chemotherapy.

## 4 Discussion

The primary strategy adjustment in our study involved strict adherence to the ASTRO guidelines for selecting eligible patients for sole IORT treatment. This change was prompted by our negative experience, which was published in 2021 [10]. In our previous study conducted using T‐IORTSCG criteria, the IORT group exhibited a significantly higher LRR than the EBRT group, with a seven‐fold greater risk in the ineligible subgroup and a 10‐fold higher risk for patients less than 50 years old [10]. The criteria for sole IORT changed significantly in April 2021, in adherence to the ASTRO guidelines. Now, eligibility requires smaller tumors (measuring less than 2 cm), positive hormone status, absence of lymph node involvement or LVI, negative resection margins, and a minimum age of 50 years. This shift differs significantly from the previous T‐IORTSCG criteria, which allowed for larger tumors measuring up to 3 cm, negative hormone status, positive LVI, and a minimum age of 40 years. After adhering to ASTRO guidelines in the current study, the eligibility for sole IORT increased from 17% (17/47) to 45% (61/137) in the IORT group. Patients in the IORT group, unlike those in the EBRT and IORT+boost groups, were characterized by a higher percentage in the suitable risk group (older age, smaller tumor size, fewer lymph node metastases) and lower rates of utilization of chemotherapy and targeted therapy. Interestingly, no statistically significant differences were observed among the three treatment groups (EBRT/IORT/IORT+boost) in various treatment outcomes (including LRR, distant metastasis, any recurrence, and mortality). A study conducted per Hoag criteria, similar to T‐IORTSCG criteria except for its requiring negative LVI, using the same IORT device (Xoft Axxent system), still resulted in a significantly higher local recurrence rate in the IORT group (5.95%) than in the EBRT group (0.5%) [11]. These results suggest that implementing different treatment strategies, specifically strict adherence to the ASTRO guidelines for selecting eligible patients for sole IORT treatment, could mitigate negative outcomes to favorable ones.

The ASTRO guidelines encompass a spectrum of factors, including patient‐related elements (like age), disease‐specific factors (such as tumors measuring less than 2 cm, positive hormone status, absence of lymph node involvement, and LVI), and treatment‐associated considerations (ensuring negative resection margins). Our study revealed different outcomes associated with distinct factors that were statistically significant. For instance, the pN stage emerged as a significant predictor of distant metastasis, whereas the section margin status, age, and pN stage were identified as statistically significant predictors of total deaths. Significant factors for event recurrence included radiotherapy type, section margin status, and ER/PR status. In the context of local‐regional recurrence, statistical significance was noted for these factors, along with LVI status. When choosing patients for exclusive IORT, adherence to criteria from studies such as the TARGIT‐A trial, ELIOT trial, American Brachytherapy Society consensus [12], European Society for Radiotherapy and Oncology (ESTRO) guidelines [13], and Hoag's criteria necessitated meeting specific conditions, including the minimum age, maximum tumor size, absence of lymph node involvement, and negative section margins [5, 6, 9]. Although a positive hormone status and negative LVI are required in ASTRO guidelines, they are not mandatory criteria for other protocols. Notably, none of the mentioned criteria require positive hormone receptor status. However, negative LVI is required only in the American Brachytherapy Society consensus and Hoag's criteria. Per the hazard ratio analysis, patients with the positive ER/PR hormone receptor status exhibited a lower risk of LRR than those with the negative status. Additionally, positive LVI increased the risk of LRR approximately four‐fold compared with negative LVI. Our findings underscore the importance of adhering to the ASTRO 2017 guidelines, which exclude cases with negative hormone receptor status and positive LVI for sole IORT. In an analysis of three prospective trials focusing on brachytherapy for accelerated partial breast irradiation, the estrogen receptor‐negative status emerged as the sole factor significantly associated with breast recurrence in a cohort of over 1900 patients [14]. A Meta‐regression analysis also concluded that the balance between the benefit and risk of partial breast irradiation, including IORT, appears optimal for women with smaller hormone receptor‐positive tumors [15]. The prevalence of LVI in Taiwan, as indicated in this study (36.3% of 583 cases) and another study (21.4% of 1640 cases), appears to be considerably higher than that reported in Western countries, such as the TARGIT‐A trial [6] (10.8% of 3451 cases) and another study [15] (12.7% of 77,425 women). LVI was significantly associated with poor local control and poorer overall survival [16, 17]. In a study of 77,425 women with early breast cancer, 12.7% had LVI, which was associated with poor grade, high 21‐gene recurrence scores (26–100), and positive lymph nodes (all *p* < 0.001). In multivariate analysis, LVI was linked to worse overall survival in node‐negative patients (HR 1.37) [16]. A meta‐analysis of 15 studies involving 21,704 patients found that LVI is associated with poorer overall survival (HR = 1.46), increased risk of distant metastases (HR = 2.08), and higher local recurrence rates (HR = 2.00) [17].

Unlike unpredictable disease‐related factors and nonmodifiable patient‐related factors, both radiotherapy type and section margin status represent essential, modifiable treatment factors capable of enhancing treatment outcomes. The responsibility for achieving a negative section margin lies with the surgeon whenever anatomically feasible. Exclusion criteria for sole IORT were implemented to ensure patient safety and treatment efficacy. After gathering comprehensive pathological information, it is crucial to evaluate the need for supplemental radiotherapy in patients not meeting the ASTRO 2017 guideline criteria for sole IORT.

In carefully selected low‐risk patients, full‐dose IORT was considered effective as standard EBRT for in‐breast recurrence [7, 13]. The reliability of the ASTRO guideline in selecting suitable patients for sole IORT is noteworthy. Per the ASTRO criteria, a study revealed that the 5‐year ipsilateral breast recurrence rates were 1.5% in the suitable group, 4.4% in the cautionary group, and 8.8% in the non‐suitable group, and these rates differed significantly from each other [18]. Among patients classified as suitable according to the ASTRO 2017 guidelines, no recurrent events or cancer‐related deaths were observed. Additionally, recurrence events were significantly higher in the non‐suitable group than in the suitable group. The cumulative probability of LRR differed significantly between the suitable and non‐suitable groups, both overall and within patients receiving sole IORT. Overall, the 5‐year recurrence rate was 0.0% for the suitable group and 4.6% for the non‐suitable group. Within the IORT subgroup, the 5‐year recurrence rate was 0.0% for the suitable group and 8.7% for the non‐suitable group. Interestingly, when focusing on patients receiving standard radiotherapy (the EBRT and IORT+boost groups), no significant differences were found between the suitable and non‐suitable groups. The Hoag Memorial Hospital study also concluded that standard adjuvant radiotherapy (incorporating supplementary EBRT for high‐risk patients) had the potential to eliminate significant differences between ASTRO risk groups. In the cohort of 1350 patients treated with IORT only, the ASTRO category curves of recurrence risk were distinct (5‐year recurrence risk: suitable 4.19%; cautionary 6.13%; unsuitable 11.31%; *p* = 0.004). However, within the entire sample of 1600 patients, supplementary EBRT was administered to those deemed high‐risk, and none of the curves exhibited statistically significant differences [11]. These findings emphasize the effectiveness of the ASTRO IORT guidelines as a valuable tool for guiding risk‐adapted treatments.

The ASTRO guidelines suggest that non‐suitable patients might benefit from standard radiotherapy. Within the non‐suitable group, the LRR rate was significantly higher in the IORT group than in the EBRT and IORT+boost groups. Applying sole IORT outside ASTRO‐suitable patients revealed higher risks of recurrence. When treated with IORT only, the 5‐year probability of local recurrence was 8.7% in our 76 patients categorized as ASTRO non‐suitable (comprising cautionary and non‐suitable). At Hoag Memorial Hospital, the 5‐year probability of local recurrence was 6.13% in 496 ASTRO‐cautionary patients and 11.31% in 150 ASTRO‐unsuitable patients [11]. Notably, patients in the IORT group had lower rates of utilization of chemotherapy and targeted therapy than those in the EBRT and IORT+boost groups. The higher risk of LRR observed in the IORT group may raise questions about the impact of weaker adjuvant systemic treatment. However, after adjusting for clinical, pathological, and treatment factors, the sole IORT group showed a significantly higher risk of LRR than the EBRT group. In both non‐suitable patients and the overall patient population, there was approximately a five‐fold increased risk of LRR with sole IORT compared with EBRT. These findings align with those of prior studies, including our previously published paper [10, 19, 20]. Two meta‐regression studies found that compared with EBRT, IORT was associated with higher odds of 5‐year local recurrence, approximately 2–3 times higher [9, 15]. IORT has proven its efficacy as a de‐escalating adjuvant therapy for low‐risk patients; however, it might not adequately serve those deemed unsuitable. The comparative efficacy of the initial intent‐to‐treat IORT versus EBRT tumor bed boost dose for local control and side effects remains under investigation and will not alter the policy [21, 22, 23]. Hence, patients initially scheduled for sole IORT but deemed unsuitable based on final pathology should consider supplemental EBRT. A study demonstrated that patients deemed unsuitable for IORT alone without receiving recommended risk‐adapted EBRT had an ipsilateral breast tumor recurrence rate of 4.7%, compared with 1.7% for patients who were either suitable for IORT alone or deemed unsuitable but received adjuvant EBRT, with a median follow‐up period of 5.1 years [24]. The TARGIT‐R trial also demonstrated that the 5‐year Kaplan–Meier recurrence risk was 8% for patients who received IORT and only 1.2% for patients who received IORT with supplementary EBRT [25]. Therefore, supplemental EBRT should be considered for patients initially programmed for sole IORT but deemed unsuitable based on their final pathological results.

**FIGURE 4:**
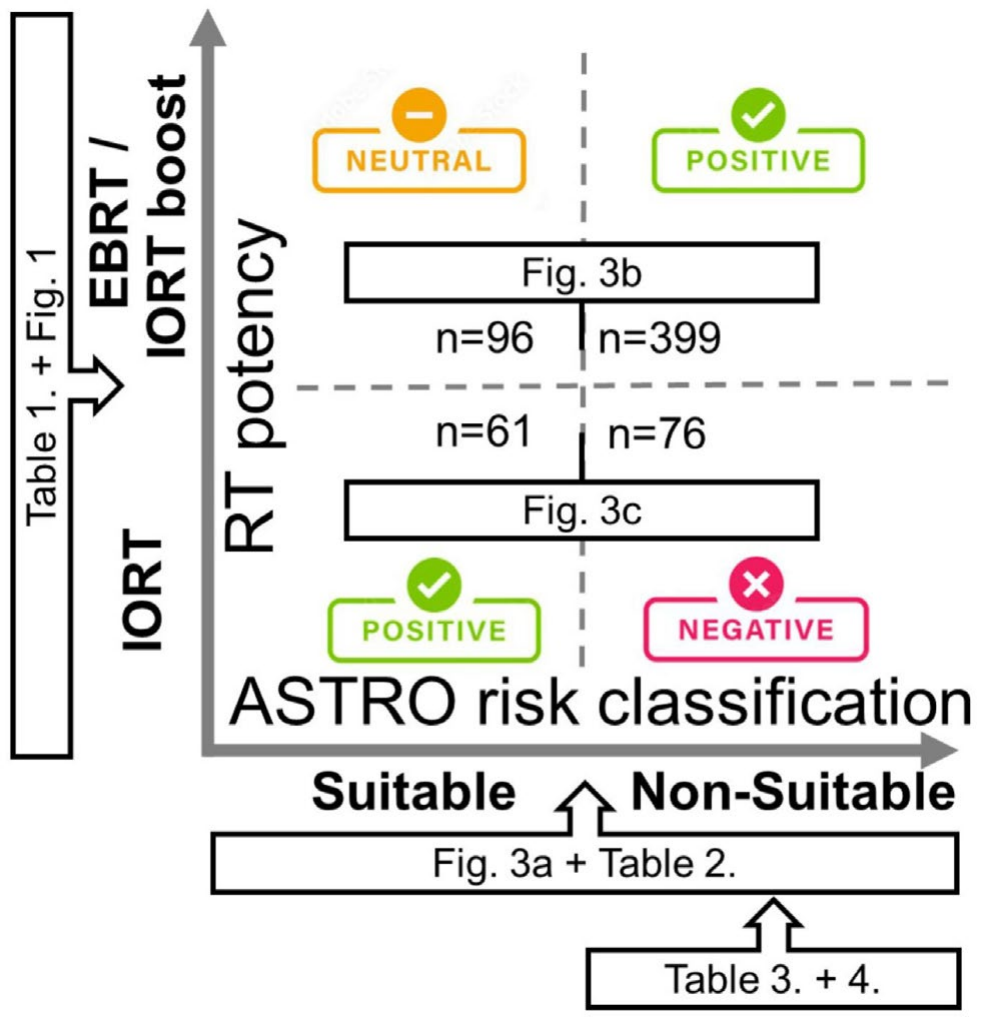
Influence of RT potency and ASTRO risk classification on clinical outcomes diagram. ASTRO risk classification on the X‐axis, RT potency on the Y‐axis.

The essence of this study is illustrated in Figure 4. The ASTRO risk category is a factual aspect, whereas the choice of adjuvant radiotherapy potency is considered subjective. Decisions need to be grounded in factual information. Positive outcomes were observed when low‐potency treatment was administered to low‐risk patients and high‐potency treatment was provided to high‐risk patients. Conversely, negative outcomes are noted when low‐potency treatment is administered to high‐risk patients. The perspective of each table and figure is also indicated. The current risk‐adapted strategy has demonstrated notable enhancements in outcome variables. However, it is important to recognize the limitations inherent in our study, such as its retrospective nature, single‐center focus, nonrandomized design, and the relatively small number of patients included in it. The ASTRO 2017 guidelines outline specific pathologic risk factors, such as requiring a section margin of at least 2 mm, considering the invasive lobular cancer type, and assessing for extensive intraductal components. Unfortunately, our study did not incorporate these detailed analyses, which could impact the comprehensive assessment of outcomes and risk stratification. Nevertheless, the findings from our study still advocate strict adherence to the protocols outlined in the ASTRO 2017 guidelines for accelerated partial breast irradiation involving sole adjuvant IORT. Altering treatment policies in response to negative experiences signifies remarkable progress. However, further data collection and extended follow‐up periods are warranted in the future to strengthen these conclusions.

Implementing sole adjuvant IORT for breast cancer patients under the ASTRO 2017 protocols effectively addressed previous negative outcomes. Adhering to patient selection based on ASTRO guidelines resulted in no statistically significant differences across all treatment outcomes among the three distinct treatment groups (EBRT/IORT/IORT+boost). The risk of LRR was approximately five times higher with sole IORT than with EBRT. Therefore, while IORT serves as a de‐escalating adjuvant therapy for low‐risk patients, its suitability for certain cases is vital. The ASTRO guideline proved reliable in selecting suitable patients for sole IORT because no recurrent events or cancer‐related deaths were observed in suitable patients. However, for patients initially programmed for sole IORT but deemed unsuitable based on final pathological results, supplemental EBRT emerged as an effective rescue treatment. This approach maximizes the benefit of IORT in suitable patients while mitigating the LRR risk in unsuitable patients through supplementary EBRT. These findings underscore the effectiveness of the ASTRO IORT guidelines as a valuable tool for guiding risk‐adapted treatments.

## Author Contributions

**Hsin‐Yi Yang:** conceptualization (equal), data curation (equal), methodology (equal), writing – original draft (equal). **Yuk‐Wah Tsang:** conceptualization (equal), methodology (equal), writing – review and editing (equal). **Chi‐Wen Tu:** conceptualization (equal), supervision (equal), writing – review and editing (equal). **Yu‐Chen Hsu:** conceptualization (equal), data curation (equal), formal analysis (equal), supervision (equal), writing – original draft (equal), writing – review and editing (equal).

## Conflicts of Interest

The authors declare no conflicts of interest.

## Data Availability

The data that support the findings of this study are available from the corresponding author upon reasonable request.
